# Comprehensive Approach to the Interpretation of the Electrical Properties of Film-Forming Molecules

**DOI:** 10.1021/acs.jpcb.2c04526

**Published:** 2022-09-02

**Authors:** Anna Chachaj-Brekiesz, Jan Kobierski, Rosa Griñón Echaniz, Anita Wnętrzak, Patrycja Dynarowicz-Latka

**Affiliations:** † Faculty of Chemistry, Jagiellonian University, Gronostajowa 2, 30-387 Kraków, Poland; ‡ Department of Pharmaceutical Biophysics, Faculty of Pharmacy, Jagiellonian University Medical College, Medyczna 9, 30-688 Kraków, Poland

## Abstract

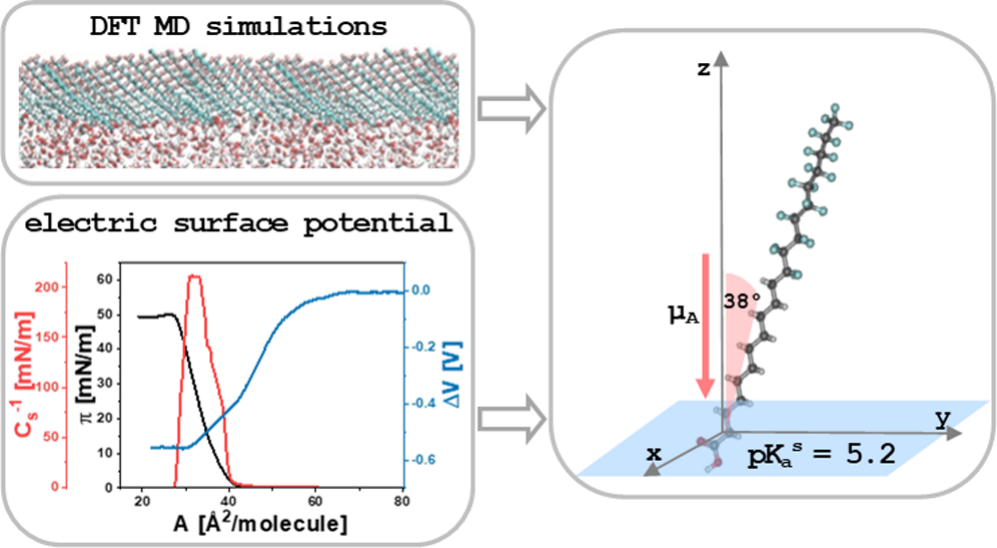

This paper presents a general protocol for the interpretation of the electric surface potential of Langmuir monolayers based on a three-layer capacitor model. The measured values were correlated with the results from DFT molecular dynamics simulations, and, as a result, the local dielectric permittivities and dipole-moment components of molecules organized in the monolayer were obtained. The main advantage of the developed approach is applicability to amphiphiles of any type; irrespective of the structure of the polar head as well as the molecular organization and inclination in the surface film. The developed methodology was successively applied to an atypical surface-active compound, perfluorodecyldecane, and its derivatives containing the hydroxyl, thiol, and carboxyl moiety. The following contributions to the apparent dipole moments connected with the reorientation of water molecules and local dielectric permittivities in the vicinity of polar and apolar molecule parts, respectively, were determined: μ^w^/ε_w_ = −0.85 D, ε_p_ = 5.00, and ε_a_ = 1.80. Moreover, the investigated perfluorodecyldecane derivatives were comprehensively characterized in terms of their surface activity, film rheology, and effective surface dissociation equilibria. The proposed methodology may be crucial for the process of the design and the preliminary characterization of molecules for sensor and material science applications.

## 1 Introduction

**Figure 1:**
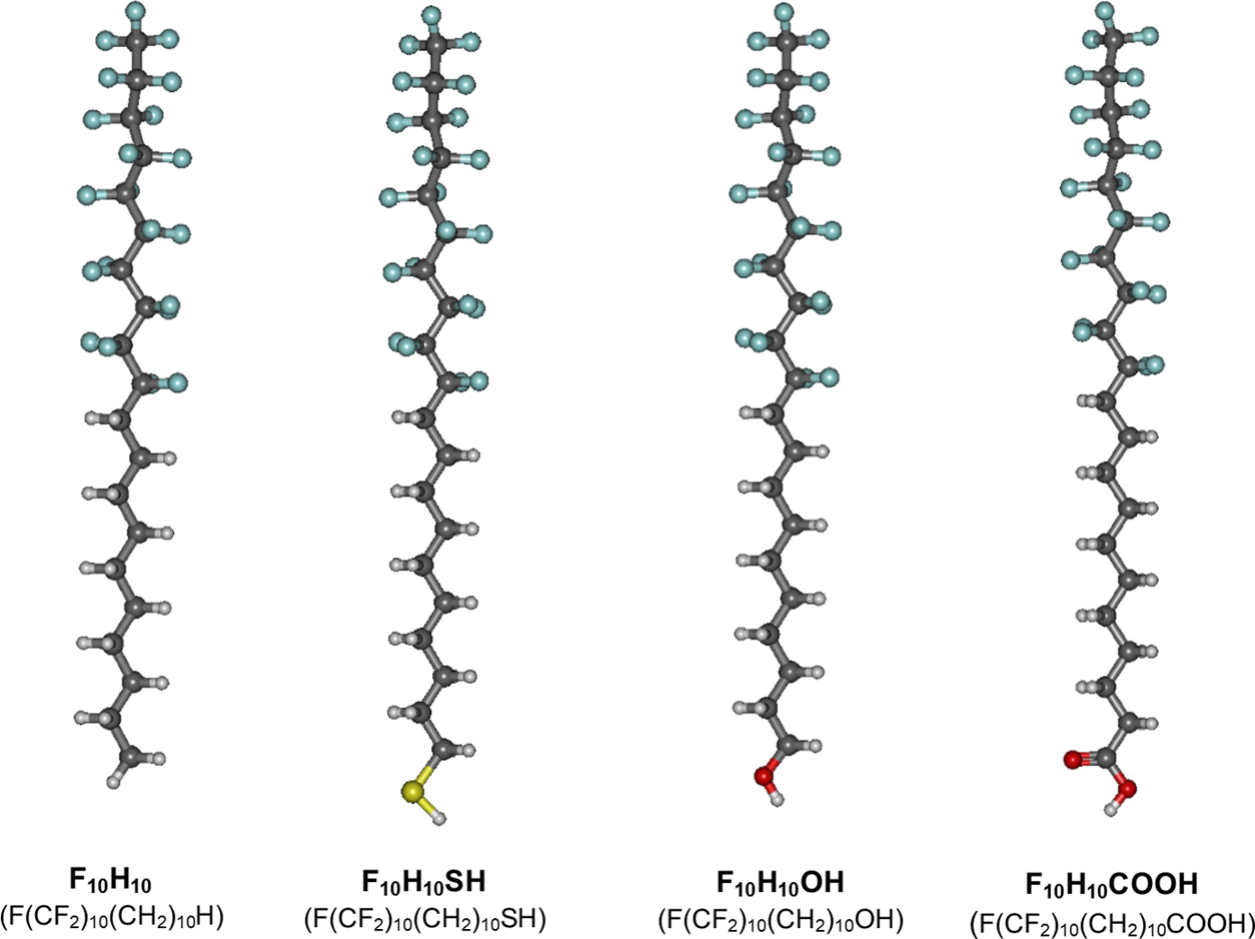
Molecular structures of the investigated molecules optimized with the DFT method using Gaussian software.

The classical method widely used to characterize monomolecular layers formed at the air/water interface (Langmuir monolayers)^1^ is based on the measurement of surface pressure–area (π–*A*) isotherms. Analysis of the obtained π–*A* experimental curves provides first-hand information on the ability of the studied molecules to form stable monolayers, allows to determine (i) their characteristic parameters (such as molecular area), (ii) the monolayer physical state (based on the compressibility modulus *C*_s_^–1^), and (iii) collapse behavior. In addition, in the case of multicomponent monolayers, information on the mutual miscibility of film components and their interactions can be obtained. Apart from the classical surface manometry, many complementary techniques for *in situ* studies of monolayers were developed, involving microscopic^2^ (fluorescence or polarizing microscopes), spectroscopic^3^ (e.g., PM-IRRAS, SFG), and X-ray scattering^4^ (e.g., GIXD) methods. The electric surface potential change (Δ*V*) measurements are equally important, although less frequently used. Although Δ*V* quantity meets the additivity rule and can be used to characterize miscibility and interactions between molecules in multicomponent systems,^5,6^ it is mainly applied to investigate one-component monolayers. In this context, it enables the determination of the electrical parameters (such as dipole moments and electrical permittivity) of the molecules in the surface layer,^7^ tracks changes in the molecular orientation during compression, and follows the formation of multilayers and domains.^8,9^ Such information obtained for thin, organized, and defectless films is especially desired when considering applications of new compounds in biomaterials, nanoelectronics, and sensorics.^1,10^ Although surface potential measurements have been performed for decades, theoretical models for relating experimental Δ*V* values to the ordering and structure of molecules in a film remain unchanged since the 1980s. Generally, an amphiphilic compound can be perceived as an electric dipole. Amphiphiles organized in the uniform monolayer at the air/water surface adopt characteristic orientation (the polar head is anchored in the water while the hydrophobic chains protrude to the air), which results in a charge gradient that is perpendicular to the surface. Thus, for the nonionized monolayer, the measured electric surface potential change Δ*V* is related to the normal (to the surface) component of the dipole moment of film molecules μ_⊥_
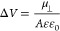
1where ε is the relative permittivity (dielectric constant) of the monolayer, ε_0_ is the permittivity of vacuum, and *A* is the average area per molecule at the surface. The unknown ε value is often included in the so-called apparent dipole moment^11^ of molecule in the film
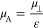
2Theoretical models of surface potential (two-^12^ or three-layer^13,14^ capacitors, reviewed in detail in refs (7, 15)) are based on the Helmholtz equation,^16^ where a monolayer is treated as a parallel-plate condenser comprising an array of uniformly distributed dipoles. In the most frequently used approach (based on the three-layer capacitor model; Demchak and Fort model^14^), μ_A_ can be expressed as a sum of three contributions
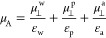
3In the above equation, ε_p_ and ε_a_ are local relative permittivities for polar and apolar parts of film molecules, respectively; μ_⊥_^p^ and μ_⊥_^a^ are the components of dipole moment normal to the water surface for polar and apolar groups, respectively; while μ_⊥_^w^/ε_w_ is the contribution from the reorientation of water molecules. In the standard methodology,^17,18^ surface electric potential change measurements are performed for the selected compounds bearing the same apolar parts and different polar parts, or vice versa. Then, the group dipole moments (μ_⊥_^p^, μ_⊥_^a^) are calculated based on the structure of each amphiphile by adding up the bond dipole moments and taking into account the angles between them. The relative permittivity values (ε_p_, ε_a_) are obtained by solving in pairs equations of type (3) for molecules of the same apolar parts and different polar parts, or vice versa. It is assumed that the contribution from the reorientation of water molecules for each pair of equations is the same. The described approach was improved by introducing DFT-optimized molecular conformations to determine μ_⊥_^p^ and μ_⊥_^a^ values; additionally, multiple linear regression was implemented to obtain ε_p_ and ε_a_ constants.^19^ However, this methodology still has some limitations. First, it assumes the vertical orientation of the molecules at the air/water interface in their most packed arrangement, while the molecules remain slightly inclined at this state in some cases.^20^ Second, for nonclassical film-forming molecules with a nonamphiphilic structure, devoid of the typical polar group (for example, semifluorinated hydrocarbons), the differentiation between the polar and apolar parts of the molecule is problematic. Therefore, the aim of this research is to present a universal model that can be applied to molecules of any structure, based on DFT modeling. For our studies, we have selected a series of semifluorinated molecules, which have attracted a lot of attention in recent years due to their interesting properties and diverse applications.^21^ These hybrid molecules comprise two incompatible fragments in their structure, hydrogenated and perfluorinated moieties,^20,22−26^ and exhibit peculiar behavior. The most striking difference, compared to alkanes and perfluoroalkanes, is their surface activity^25−27^ and liquid–crystalline properties,^25,26,28^ which are absent in the counterpart apolar molecules. A plethora of papers on the physicochemical and structural properties of semifluorinated alkanes, including surface micelles formation, have appeared in the literature (for reviews, see^25,26,29^ and references therein). However, the electrical properties of films of amphiphilic molecules containing a perfluorinated core attached to different polar groups have not been systematically studied. For our investigations, we have chosen 1,1,1,2,2,3,3,4,4,5,5,6,6,7,7,8,8,9,9,10,10-henicosafluoroeicosane (F(CF_2_)_10_(CH_2_)_10_H abbr. F_10_H_10_) and its amphiphilic derivatives containing different polar groups, namely, F_10_H_10_SH, F_10_H_10_OH, and F_10_H_10_COOH. The structures of the studied molecules are shown in Figure 1.

## 2 Experimental and Theoretical Methods

### 2.1 Materials

The following compounds were investigated in this study: 1,1,1,2,2,3,3,4,4,5,5,6,6,7,7,8,8,9,9,10,10-henicosafluoroeicosane (F(CF_2_)_10_(CH_2_)_10_H, abbr. F_10_H_10_), 11,11,12,12,13,13,14,14,15,15,16,16,17,17,18,18,19,19,20,20,20-henicosafluoro-eicosane-1-thiol (F(CF_2_)_10_(CH_2_)_10_SH, abbr. F_10_H_10_SH), 11,11,12,12,13,13,14,14,15,15,16,16,17,17,18,18,19,19,20,20,20-henicosafluoroeicosan-1-ol (F(CF_2_)_10_(CH_2_)_10_OH, abbr. F_10_H_10_OH), and 11,11,12,12,13,13,14,14,15,15,16,16,17,17,18,18,19,19,20,20,20-henicosafluoroheneicosanecarboxylic acid (F(CF_2_)_10_(CH_2_)_10_COOH, abbr. F_10_H_10_COOH). All semifluorinated compounds were synthesized according to literature procedures, and their analytical data were in agreement with those previously reported in ref (30) (for F_10_H_10_) and ref (20) (for F_10_H_10_SH, F_10_H_10_OH, and F_10_H_10_COOH). For Langmuir monolayer experiments each of the investigated compounds was dissolved in spectral grade chloroform (Sigma-Aldrich) with a typical concentration of 0.2–0.3 mg/mL. Deionized ultrapure water from a Millipore system with a resistivity of 18.2 MΩ cm and pH 5.6 was used in Langmuir experiments. The sodium chloride solutions of concentrations of 0.1, 0.01, and 0.001 mol/dm^3^ were prepared by dissolving solid sodium chloride (Sigma-Aldrich, purity >99%) in ultrapure water. The hydrochloric acid solution with a concentration of 0.001 mol/dm^3^ was prepared by diluting the HCl standard solution (ChemPur) with ultrapure water.

### 2.2 Langmuir Monolayer Characterization

Surface pressure–area (π–*A*) and electrical surface potential change–area (Δ*V*–*A*) curves were measured simultaneously using NIMA equipment: a two-barrier trough of the total area of 600 cm^2^ (612D) coupled with the surface pressure and surface potential sensor. In a typical experiment, 50–100 μL of the investigated compound solution in chloroform was carefully spread with a microsyringe onto the subphase surface. After solvent evaporation (approximately 10 min), the film was compressed with a barrier speed of 20 cm^2^/min. During the experiments, the subphase temperature was maintained at 20 ± 0.1 °C by the Julabo thermostat. Surface pressure was recorded with an accuracy of ±0.1 mN/m using a Wilhelmy plate made of chromatography paper (Whatman Chr1) immersed in the subphase as the pressure probe. During electrical surface potential change measurements, the vibrating plate was located around 2 mm above the subphase surface while the reference electrode was placed in the subphase. The electrical surface potential was registered with an accuracy of ±15 mV and ±2 Å^2^/molecule. The surface pressure–area and electric surface potential change–area isotherms presented here are representative curves selected from at least two overlapping experiments.

### 2.3 Theoretical Calculations

The dipole moments were calculated for previously geometrically optimized systems using the Gaussian 16 software package.^31^ Geometry optimization was performed by density functional theory (DFT) modeling. All calculations were performed with the B3LYP functional,^32,33^ a basis set including diffuse and polarization functions, i.e., 6-311++G(3df,3pd)^34,35^ without damping. Systems were optimized with the default UltraFine integration grid, default integral cutoffs, and a combination of EDIIS and CDIIS tight convergence procedures, with no Fermi broadening. The dipole moments of polar μ_⊥_^p^ and apolar μ_⊥_^a^ parts of a molecule were determined utilizing the quantum theory of atoms in molecules (QTAIM) in AIMAll software.^36^

Molecular dynamics calculations were performed in the Amber20 package.^37^ Each analyzed system consisted of two symmetric rectangular monolayers, each having 128 perfluorodecyldecane or its derivative molecules, separated by 30,000 water molecules. Simulated systems were prepared in Packmol software.^38^ The General AMBER Force Field 2 (GAFF 2) was used, with the partial atomic charge calculated by Gaussian 16 using the Hartree–Fock method and the 6-31G(d) basis set. Periodic boundary conditions were utilized. The TIP3P model^39^ was used to simulate water molecules. The energy of the systems was minimized by 50,000 steps. The systems were equilibrated by 75,000 steps with a 0.001 ps timestep, followed by 300,000 steps with a 0.002 ps timestep. Production calculations were carried out in the isothermal–isobaric ensemble with the constant surface tension of 30 mN/m (NPγT) and with a 0.002 ps timestep. The temperature was set at 293 K and the Langevin thermostat was used. A Berendsen aerostat was used to control pressure at 1 bar. The simulation was carried out for 500 ns, and the last 10 ns were used for the analysis. Radial pair distribution functions were determined in the Cpptraj program.^40^

## 3 Results and Discussion

### 3.1 Experimental π–*A* Isotherms

**Figure 2:**
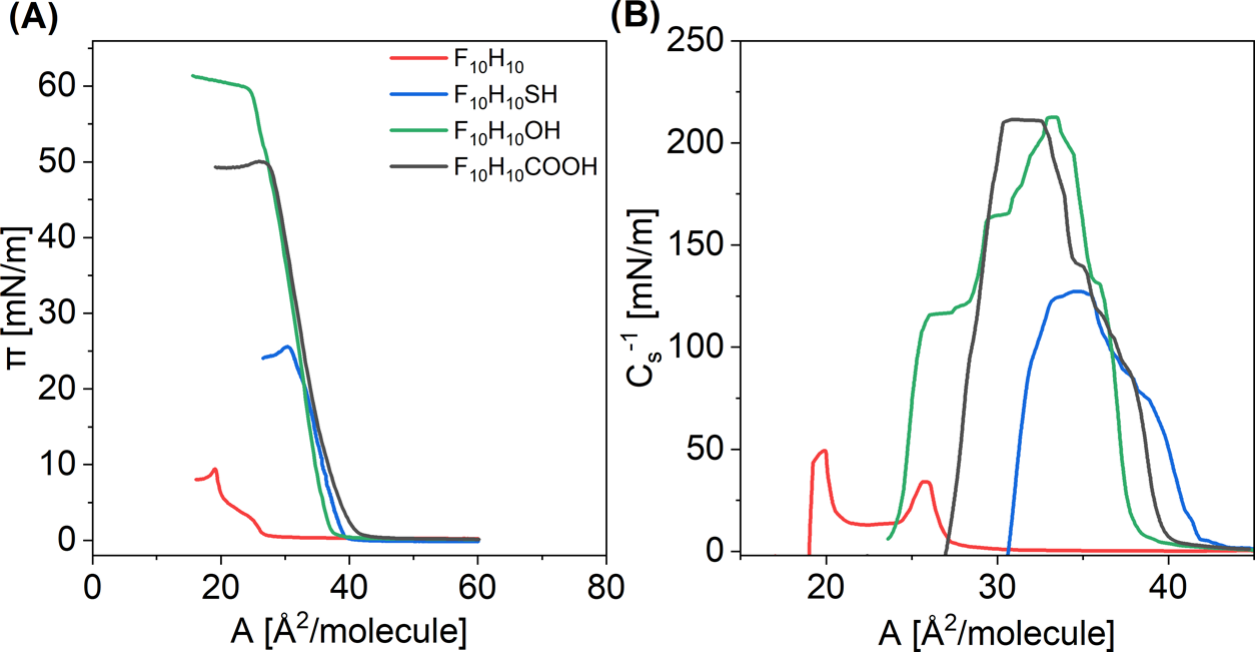
Experimental surface pressure–area isotherms (A) and calculated compressibility moduli curves (B) for monolayers of nonionized perfluorodecyldecane derivatives on 0.001 mol/dm^3^ aqueous HCl solution as a subphase at 20 °C.

In the first stage of our investigations, we looked at how the surface activity of the semifluorinated amphiphiles changes with the type of polar group. For this purpose, the surface pressure–area per molecule (π–*A*) isotherms were registered using ultrapure water and an aqueous solution of HCl (0.001 mol/dm^3^) as the subphase. The reason for using the acidic subphase was to check whether moving the equilibrium of dissociation of some ionizable polar groups toward their neutral form (i.e., −COO^–^ + H^+^ → −COOH) modifies the surface activity of the studied compounds. The comparison of Langmuir isotherms recorded at different subphases (water or NaCl solutions) is presented in Figure S1 (Supporting Information). As can be seen, the curves for F_10_H_10_, F_10_H_10_OH, and F_10_H_10_SH on water practically coincide with those recorded for the acidic subphase. In the case of F_10_H_10_COOH, the difference between isotherms is visible only at low surface pressure values (the lift off area of the isotherm recorded for the acidic subphase is ca. 2 Å greater compared to the isotherm registered for pure water). Therefore, it can be concluded that the surface activity of perfluorodecyldecane and its amphiphilic derivatives is hardly influenced by the degree of dissociation. To analyze how the surface activity of perfluorodecyldecane derivatives varies with the type of polar group, the representative isotherms recorded on the HCl solution together with the calculated compressibility moduli (*C*_s_^–1^) curves have been compared in Figure 2. The characteristic parameters read from the plots are summarized in Table 1. Values of *C*_s_^–1^, calculated on the basis of the experimental isotherm datapoints (applying the formula (41)), are helpful in describing the physical state of the surface film. Namely, *C*_s_^–1^ values below 25 mN/m suggest that the film is in a low-density liquid phase; the ranges of 25–50 and 100–250 mN/m are characteristics of the liquid expanded and liquid condensed states, respectively, while the film is in the solid state for *C*_s_^–1^ above 500 mN/m.^1^

**Table 1:** Selected Parameters Read from the Surface Pressure–Area and Electric Surface Potential Change–Area Experimental Curves Measured on 0.001 mol/dm^3^ Aqueous HCl Solution as a Subphase at 20 °C, Together with the Experimental Apparent Dipole Moment Values μ_A_^exp^ Calculated from eq 3

compound	*A*_0_^a^ (Å^2^/molecule)	*A*_c_^b^ (Å^2^/molecule)	max *C*_s_^–1 ^c (mN/m)	*A*_max_^d^ (Å^2^/molecule)	Δ*V*_max_^e^ (V)	μ_A_^exp^ (D)
F_10_H_10_	27.8	38.8	49	19.39	–0.221	–0.114
F_10_H_10_SH	41.9	47.0	127	34.99	–0.521	–0.484
F_10_H_10_OH	41.0	54.0	213	32.90	–0.579	–0.506
F_10_H_10_COOH	43.4	66.4	212	30.39	–0.554	–0.447

a The value of the area per molecule corresponding to the π–*A* isotherm’s lift off (lift off point).

b The value of the area per molecule corresponding to the beginning of the decrease in the Δ*V*–*A* isotherm (critical area).

c The maximum values of the compressibility moduli.

d The area per molecule corresponding to the maximum value of the compressibility moduli.

e The electric surface potential corresponding to the maximum packing of monolayer molecules.

The π–*A* isotherm of F_10_H_10_ starts to rise at the molecular area below 27.8 Å^2^ and collapses at an area of 19.0 Å^2^/molecule and a surface pressure of 9.5 mN/m. The isotherm shows a clear inflection above the surface pressure of 3 mN/m, which is also clearly visible in the *C*_s_^–1^–*A* plot (as a minimum), and the calculated compressibility moduli values suggest that the inflection is due to the phase transition within liquid state.^11,24^ The introduction of a hydrophilic group in the terminal position of the perfluorodecyldecane moiety strikingly influences the surface activity of the compounds obtained. Namely, compared to F_10_H_10_, isotherms of its amphiphilic derivatives show some common features: (i) the lift off areas are shifted to larger values (above 41 Å^2^/molecule), (ii) the slope of the curves is more vertical and without visible inflections (the monolayers are in a liquid condensed state), (iii) the collapse pressure is significantly higher. Nevertheless, the analysis of the π–*A* curves also shows some differences between the curves recorded for functionalized perfluorodecyldecanes. F_10_H_10_SH forms slightly more expanded films compared to F_10_H_10_OH and F_10_H_10_COOH. This is because the measured π–*A* dependencies for F_10_H_10_SH start to increase at larger areas (approximately 41.9 Å^2^/molecule) than for F_10_H_10_OH and the slope of the isotherm is more inclined than for F_10_H_10_OH and F_10_H_10_COOH. It is also reflected in the calculated compressional moduli values, which are almost 1.7 times smaller than those for F_10_H_10_OH and F_10_H_10_COOH; however, the physical state of the monolayers remains the same (liquid condensed). Furthermore, the collapse pressure of F_10_H_10_SH film is quite low (approximately 25.4 mN/m). The F_10_H_10_OH and F_10_H_10_COOH isotherms have a similar slope (and similar maximum values of *C*_s_^–1^); however, the collapse pressure value of F_10_H_10_OH is higher than that of F_10_H_10_COOH (59.8 vs 50.0 mN/m).

### 3.2 Experimental Δ*V*–*A* Isotherms

In the next step, we examined the electrical properties of surface films formed by perfluorodecyldecane and its derivatives at the air–water boundary. Initially, the electric surface potential change–area (Δ*V*–*A*) isotherms were measured for films formed on the ultrapure water as well as on 0.001 mol/dm^3^ aqueous HCl solution (Figure S2, Supporting Information). It was noticed that the experimental curves registered on both subphases overlap only for F_10_H_10_. However, in the case of other compounds (F_10_H_10_SH, F_10_H_10_OH, and F_10_H_10_COOH), there is an additional contribution to the electric surface potential connected with the double-layer potential (ψ_0_). According to the Gouy–Chapman theory, the double-layer potential (ψ_0_) is a result of the dissociation of polar head groups at the water–air interphase and/or their involvement in a hydrogen-bonding network.^42^ This issue is discussed in detail in Section 3.4.

**Figure 3:**
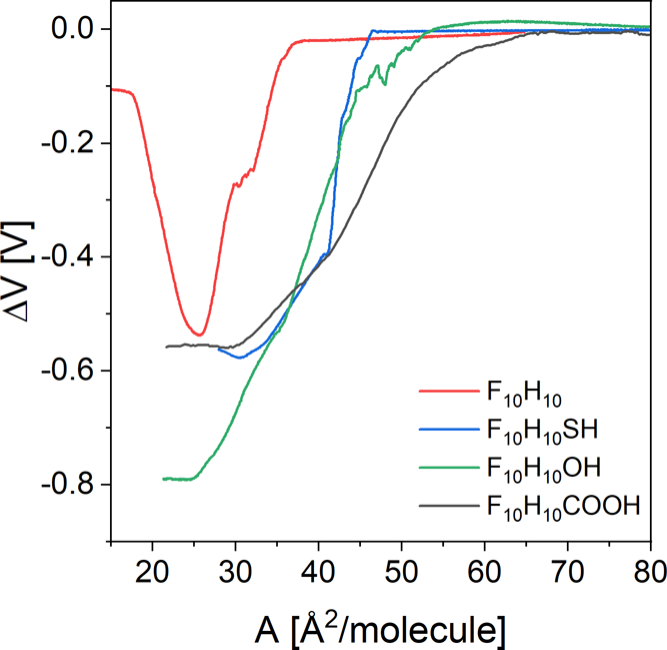
Electric surface potential change (Δ*V*)–area (*A*) isotherms measured for perfluorodecyldecane derivatives with a 0.001 mol/dm^3^ aqueous HCl solution as a subphase at 20 °C.

To determine the electric properties of film molecules resulting directly from their structure, the electric surface potential change–area (Δ*V*–*A*) curves measured for monolayers formed on 0.001 mol/dm^3^ aqueous HCl solution were used (Figure 3). In this way, the double-layer contribution (ψ_0_) resulting from different surface ionization degrees of polar groups in the investigated compounds can be avoided. The characteristic parameters read from the experimental curves are summarized in Table 1.

The Δ*V*–*A* dependencies measured for all investigated compounds are characterized by a similar course. At the beginning of compression, the Δ*V* values remain constant at approximately zero until the so-called critical area (*A*_c_)^7,43^ is reached. The value of *A*_c_ is different for each of the compounds tested and indicates the point when the hydrogen bonds with the water molecules are broken and the monolayer begins to organize.^7^ As expected, *A*_c_ has the smallest value for a purely hydrophobic molecule, F_10_H_10_, which is not involved in hydrogen bonding with water molecules. After the value of *A*_c_ upon compression is exceeded, the electric surface potential isotherm gradually decreases toward more negative values until it reaches a clear inflection point at small areas per molecule. It is interesting that for F_10_H_10_, the surface potential isotherm raises toward less negative values upon further compression. This observation, in addition to the inflection in the π–*A* isotherm, proves that the F_10_H_10_ molecules in the monolayer undergo a phase transition during compression.^11^ In addition to the critical area (*A*_c_), another key parameter that can be obtained from the experimental Δ*V*–*A* curves is Δ*V*_max_ value, which is the electric surface potential change corresponding to the closest packing in the monolayer. Δ*V*_max_ value is read from the Δ*V*–*A* curve for the molecular area *A*_max_, which corresponds to the maximum of compressibility moduli.

Values of experimental apparent dipole moments (μ_A_^exp^) can be calculated from experimental Δ*V*–*A* dependencies by applying the following equation
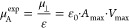
4where *A*_max_ and Δ*V*_max_ are the area per molecule and the electric surface potential corresponding to the maximum packing of the monolayer molecules. These values obtained for the studied semifluorinated molecules are summarized in Table 1.

As can be seen, the apparent dipole moment for F_10_H_10_ is small and differs from the values for amphiphilic derivatives (F_10_H_10_SH, F_10_H_10_OH, F_10_H_10_COOH). This can be explained by taking into account the molecular structure of the investigated compounds, which is discussed in the next section.

### 3.3 Interpretation of Experimental Apparent Dipole Moment Values of Monolayers Formed by Nonionized Amphiphiles

**Figure 4:**
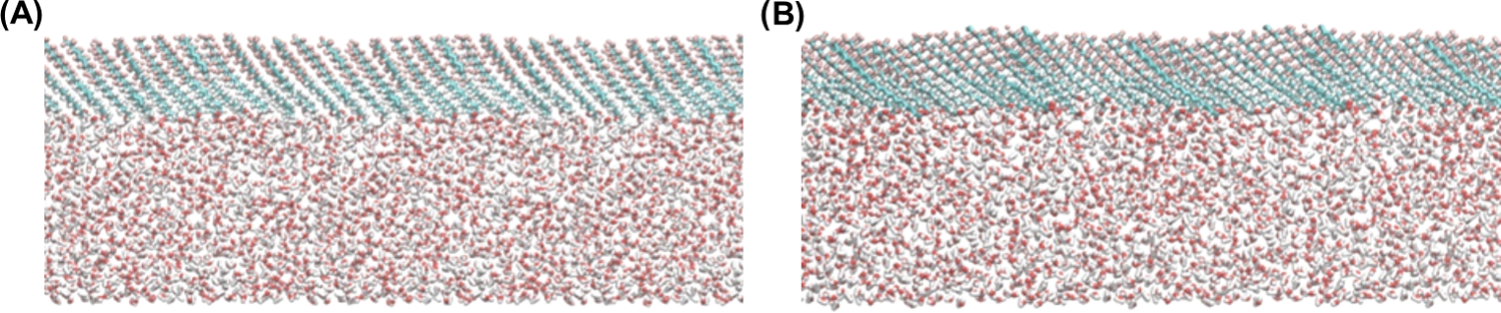
Snapshots of the exemplary monolayers at π = 30 mN/m simulated with molecular dynamics for F_10_H_10_OH (A) and F_10_H_10_COOH (B).

Taking into account the contributions to the apparent dipole moment of the film molecule (eq 3), the normal components of the dipole moment of the nonionized polar and apolar parts of the molecule (μ_⊥_^p^ and μ_⊥_^a^) can be calculated based on its structure. The standard approach involves optimization of the molecular conformation in a vacuum using semiempirical or DFT methods. The values of μ_⊥_^p^ and μ_⊥_^a^ for the polar and apolar parts of the molecule can be determined assuming that the investigated amphiphile adopts vertical orientation in a closely packed monolayer at the water–air interphase. Such an assumption was successfully applied to aromatic carboxylic acids^44^ and phosphocholines.^19^ However, for perfluorodecyldecane derivatives, this approach failed possibly because of two main reasons. First, the investigated molecules were found to be inclined in a closely packed film at the surface of the water, as shown in ref (11). Therefore, the angle between the main axis of the molecule (defined as the vector that connects the extreme positions of carbon atoms in an aliphatic chain) in the closely packed arrangement and the normal to the water–air interphase (θ) should be taken into account. Second, the precise distinction between the polar and apolar parts of the molecule may be problematic; therefore, information on the hydration shell of the hydrophilic groups is desirable. To investigate these issues, the simulations of molecular dynamics were carried out for the investigated perfluorodecyldecane derivatives with the assumption of nonionized polar groups. The obtained results confirmed that the orientation of the investigated compounds in their most packed arrangements (corresponding to max *C*_s_^–1^) is not vertical (Figure 4); the deviation of the main axis of the molecules from the normal to the water–air interphase (θ) is approximately 53° for F_10_H_10_SH, while that for the remaining studied molecules is below 47° (Table 2).

**Table 2:** Values of Normal Contributions of Dipole Moments from the Polar and Apolar Parts of the Molecules in Monolayer Calculated Using Gaussian Software

compound	θ (deg)	μ_⊥_^p^ (D)	μ_⊥_^a^ (D)	μ_A_^calc^ (D)
F_10_H_10_	47	–0.1587	0.9871	–0.103
F_10_H_10_SH	53	–0.3279	0.8261	–0.207
F_10_H_10_OH	44	–0.1411	1.0180	–0.106
F_10_H_10_COOH	38	–0.0813	0.7773	–0.329

**Figure 5:**
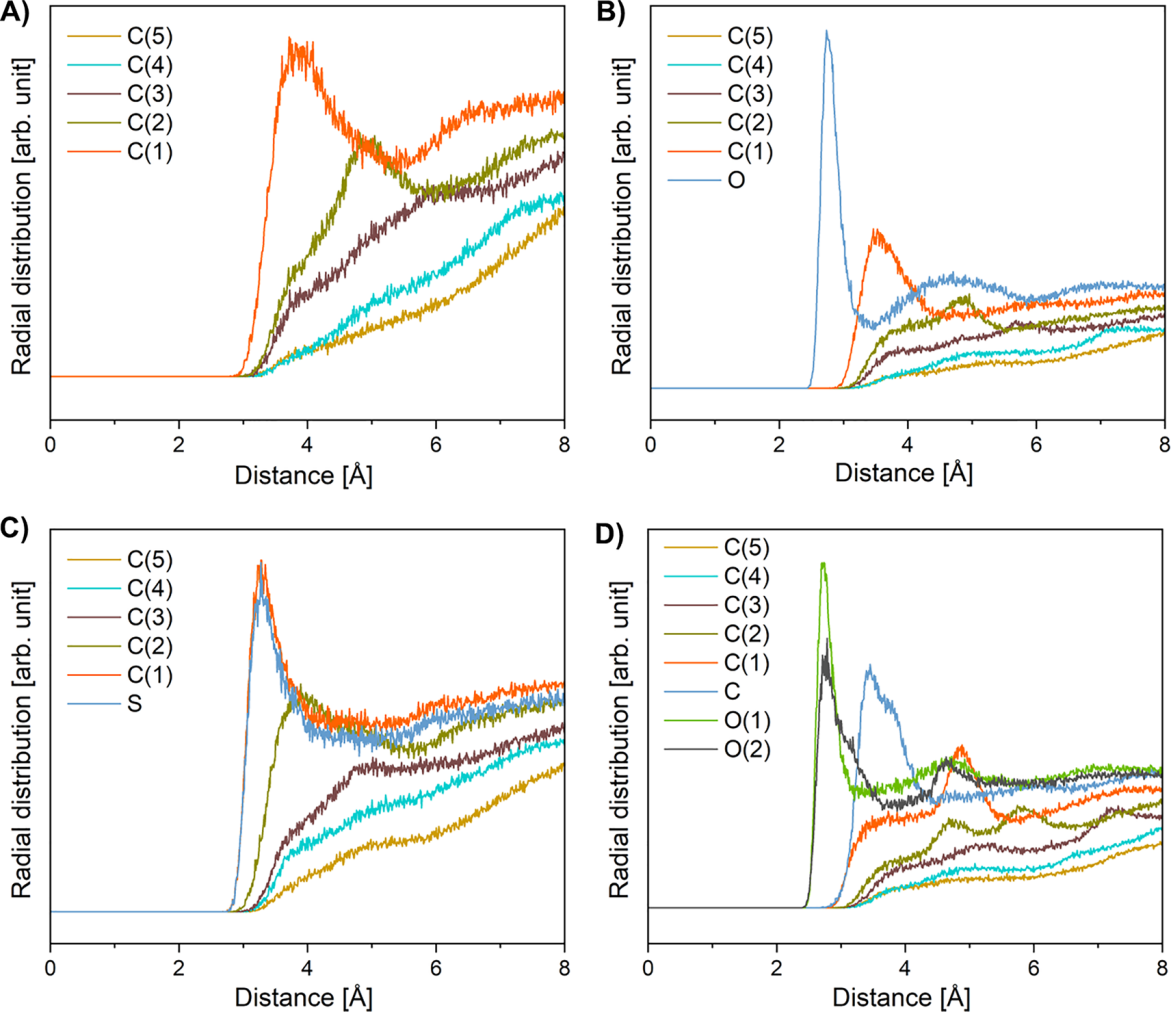
Radial distribution functions showing the average distribution of oxygen atoms from water molecules around heavy atoms within perfluorodecyldecane and its derivatives (five carbon atoms closest to the water phase and heteroatoms from adjacent polar groups): F_10_H_10_ (A), F_10_H_10_OH (B), F_10_H_10_SH (C), and F_10_H_10_COOH (D).

To identify the hydrated parts of the molecules simulated in molecular dynamics, we determined the radial pair distribution functions between carbon and oxygen atoms in the investigated molecules and oxygen atoms in water molecules (Figure 5). It was assumed that the maximum of the radial distribution function corresponding to a distance slightly above 3 Å suggests the presence of the selected atom in the first adsorbed water layer. Otherwise, a value greater than 4 Å suggests that the neighboring atom and/or further atoms in the molecule are hydrated. As it can be noticed, for each investigated compound, the distance between the first carbon atom in the hydrocarbon chain of the amphiphile and the oxygen atoms of the water molecules is clearly defined and equals approximately 3 Å. This suggests that the following groups can be perceived as hydrated (polar): −CH_3_ (in F_10_H_10_), −CH_2_SH (in F_10_H_10_SH), −CH_2_OH (in F_10_H_10_OH), and −COOH (in F_10_H_10_COOH).

Taking into account the inclination of the investigated amphiphiles at the surface in their most packed arrangements and the assignments of the hydrated parts of the molecules, the values of μ_⊥_^p^ and μ_⊥_^a^ were determined using the DFT approach (see Table 2).

In the next step, the calculated dipole moments of the polar and apolar parts of molecules (μ_⊥_^p^ and μ_⊥_^a^) were used together with experimental μ_A_^exp^ values to determine the equation on the apparent dipole moment of molecule in a monolayer using multiple linear regressions
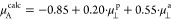
5Based on eqs 3 and 5, the average values of crucial parameters were obtained: , ε_p_ = 5.00, and ε_a_ = 1.80. The calculated local relative permittivities are slightly lower in comparison to the literature values, which lie in the range 6–7 and 2–3 for ε_p_ and ε_a_, respectively.^45^ Nonetheless, the calculated local dielectric permittivity values prove a good correlation between the experimental values of apparent dipole moments and those obtained from DFT molecular dynamics.

### 3.4 Interpretation of Experimental Apparent Dipole Moments of Monolayers from Ionized Amphiphiles

**Figure 6:**
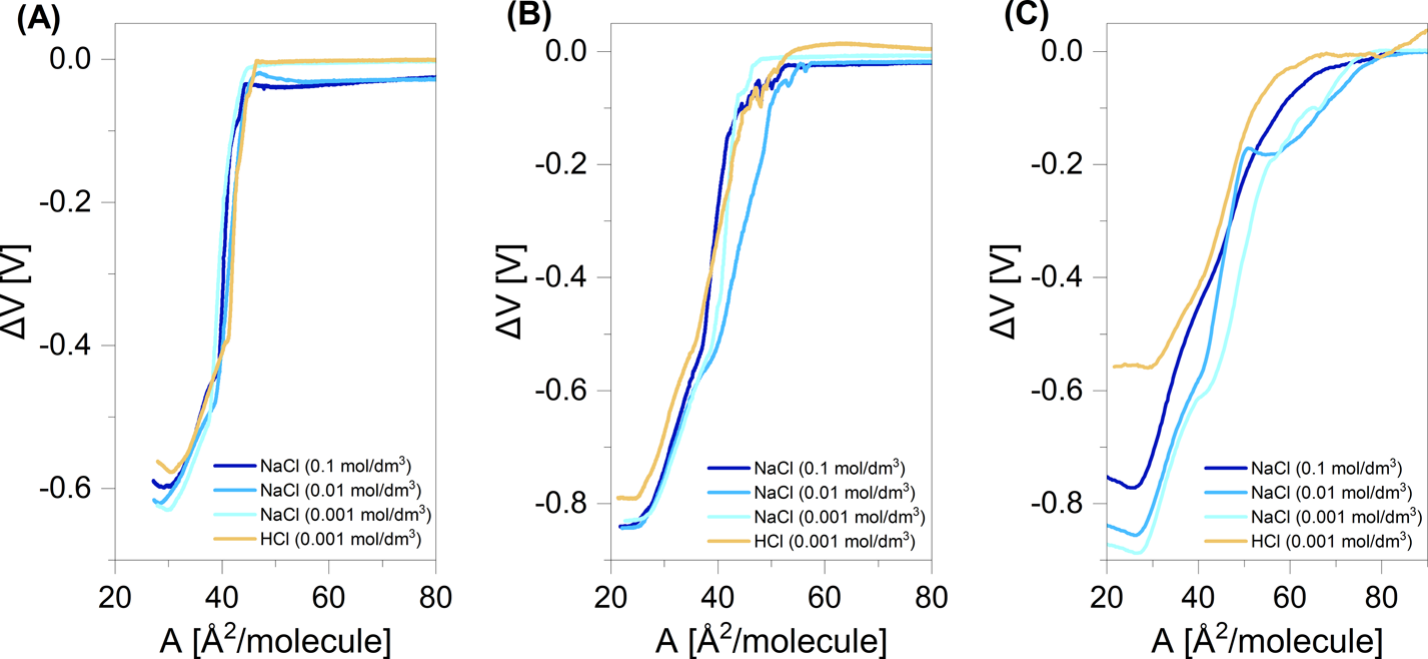
Electric surface potential change (Δ*V*)–area (*A*) isotherms measured on various subphases at 20 °C for perfluorodecyldecane derivatives: F_10_H_10_SH (A), F_10_H_10_OH (B), and F_10_H_10_COOH (C).

In the next stage of our investigations, the electrical properties resulting exclusively from polar interactions and/or H-bonding of the polar group attached to the perfluorodecyldecane moiety were analyzed. For this purpose, the Δ*V*–*A* isotherms on subphases differing in ionic strength (containing the following concentrations of NaCl: 0.1, 0.01, and 0.001 mol/dm^3^) were measured for each investigated compound. The resulting Δ*V*–*A* curves were compared with isotherms registered using an aqueous subphase containing HCl (0.001 mol/dm^3^) and are presented in Figure 6. Additionally, the Δ*V*_max_ values (corresponding to the closest monolayer packing) were read and are compiled in Table 3.

**Table 3:** Values of the Experimentally Determined Double-Layer Potential Together with the Calculated Dissociation Degree α^S^ and Mean p*K*_a_^S^ Values with Their Uncertainties Determined with Standard Deviation

compound	subphase	Δ*V*_max_ [V]	|ψ| [V]	α^S^	p*K*_a_^S^ ± Δp*K*_a_^S^
F_10_H_10_SH	HCl (0.001 mol/dm^3^)	–0.521			
	NaCl (0.001 mol/dm^3^)	–0.553	0.032	0.005	7.79 ± 0.05
	NaCl (0.01 mol/dm^3^)	–0.534	0.013	0.006	
	NaCl (0.1 mol/dm^3^)	–0.526	0.005	0.007	
F_10_H_10_OH	HCl (0.001 mol/dm^3^)	–0.579			
	NaCl (0.001 mol/dm^3^)	–0.680	0.101	0.028	6.80 ± 0.27
	NaCl (0.01 mol/dm^3^)	–0.666	0.087	0.063	
	NaCl (0.1 mol/dm^3^)	–0.643	0.064	0.113	
F_10_H_10_COOH	HCl (0.001 mol/dm^3^)	–0.554			
	NaCl (0.001 mol/dm^3^)	–0.815	0.261	0.636	5.17 ± 0.16
	NaCl (0.01 mol/dm^3^)	–0.766	0.212	0.722	
	NaCl (0.1 mol/dm^3^)	–0.714	0.160	0.812	

**Figure 7:**
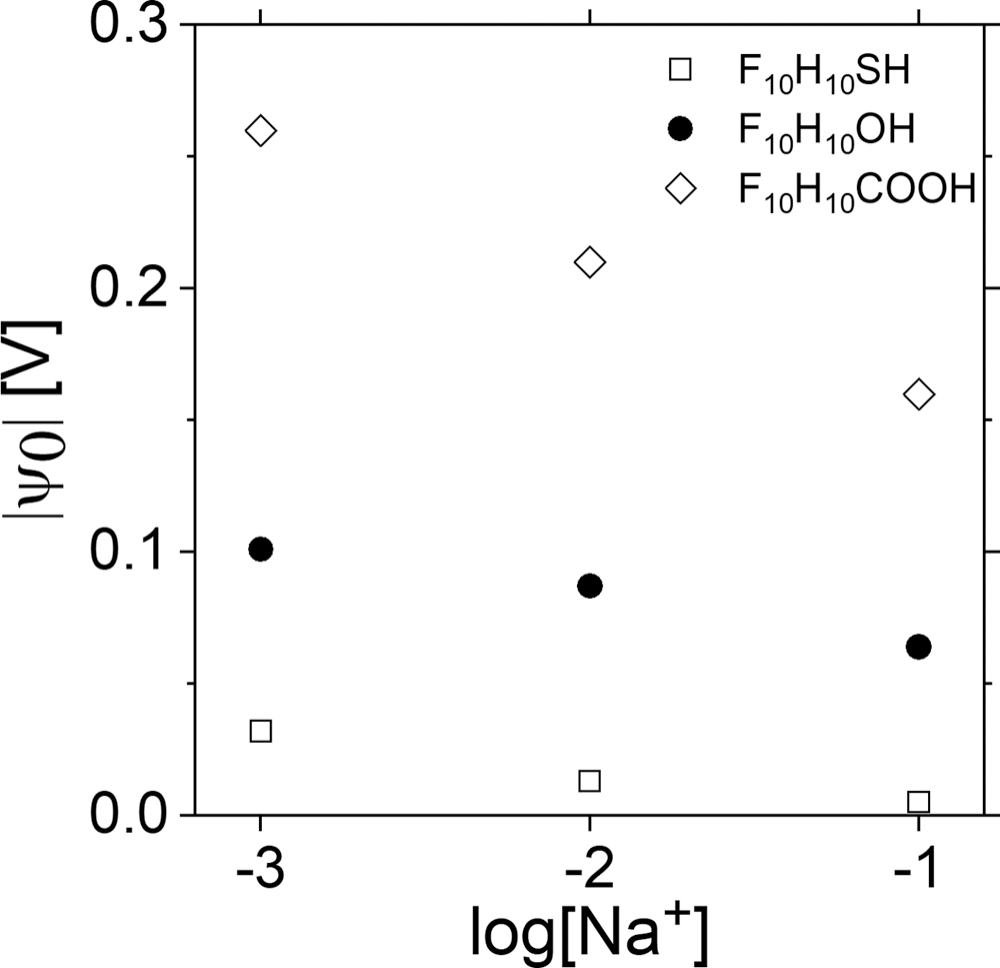
Double-layer potential as a function of the NaCl concentration in the subphase.

As it can be seen, Δ*V*_max_ values for all investigated compounds follow the same trend depending on the subphase applied. The Δ*V*_max_ values for the subphase containing 0.001 mol/dm^3^ HCl are the highest (the least negative) and correspond to the uncharged monolayer. The surface potential change for charged monolayers is the lowest (most negative) for the subphase containing 0.001 mol/dm^3^ NaCl and increases gradually as the subphase ionic strength increases. As already mentioned, for the partially charged surface layer, the electric surface potential change (Δ*V*^i^) is not only related to the molecular dipole moments but also the double-layer potential (ψ_0_) should be taken into consideration
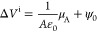
6Thus, knowing that the first segment in eq 6 corresponds to the surface potential of nonionized monolayer, ψ_0_ can be calculated
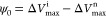
7where Δ*V*_max_^i^ and Δ*V*_max_^n^ are the electric surface potential change values corresponding to the closest packing in the ionized and nonionized monolayer, respectively. The obtained ψ_0_ values are summarized in Table 3, and their dependence on the ionic strength of the subphase is plotted in Figure 7.

From the initial analysis, it should be noticed that ψ_0_ values calculated for F_10_H_10_SH are the lowest and only one of them surpasses the experimental inaccuracy value. Nonetheless, the analysis of the data in Figure 7 shows that ψ_0_ values for all functionalized perfuorodecyldecane derivatives decrease with the increasing ionic strength. For each of the investigated compounds, the slope of this trend is different. To better understand these differences, the ionization degrees and dissociation constants of molecules at the surface were calculated using the literature methodology.^42,46^ The surface ionization degree at a temperature of 20 °C can be obtained from the following equation
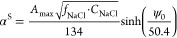
8where *f*_NaCl_ is activity coefficient value equal to 1.00, 0.90, or 0.79 for NaCl concentration of 0.001, 0.01, or 0.1 mol/dm^3^, respectively.

Finally, the p*K*_a_^S^ values were calculated using the Henderson–Hasselbalch equation
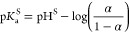
9The knowledge of p*K*_a_^S^ value is of utmost importance especially in biomedical sciences and sensorics because the majority of pivotal processes take place at the interfaces, where the value of the dissociation constant for an ionizable molecule differs from that in the bulk phase. For aliphatic (unsubstituted) carboxylic acids, bulk p*K*_a_ falls within the range of 4.7–5.0,^47^ while p*K*_a_^S^ at the air/water interface is higher. For example, the value p*K*_a_^S^ = 5.7 ± 0.2 was determined for condensed stearic acid in Langmuir monolayers from surface potential measurements.^42,48^ Similarly, for nonanoic acid, it is equal to 5.9 ± 0.6 as obtained from a combination of surface tension measurements and molecular dynamics simulations.^49^ However, it is worth to mention that in the case of amphiphiles that are slightly soluble in water (i.e., nonanoic acid), the surface adsorption/desorption equilibrium should also be taken into consideration.^49,50^ For aliphatic bases in bulk, the value reads 10.6–10.9,^47^ while it is 10.1 at the interface (determined for nonadecylamine).^51^ Similar results were obtained applying different methods, e.g., SFG.^52,53^ It is clear that at the interface there is a shift of p*K* of about 0.7 for both acids and bases toward neutral pH. This is due to the fact that the conditions at the phase boundary are more favorable for a larger accumulation of uncharged molecules, while ionized molecules avoid contact with the interface. It can be noticed that p*K*_a_^S^ values determined for amphiphilic derivatives of perfluorodecyldecane are noticeably lower than for analogous hydrocarbon derivatives, i.e., the average p*K*_a_^S^ for F_10_H_10_COOH is ca. 5.2, while that for stearic acid is 5.7. This can be explained by the electron-withdrawing effect of fluorine atoms, which causes the p*K*_a_ for fluorinated compounds to be significantly lower as compared to those of their aliphatic analogues. For example, the substitution of one hydrogen atom in acetic acid by a fluorine atom decreases p*K*_a_ from 4.8 to 2.6 in bulk.^47^ Similarly, the p*K*_a_ value for ethanol is equal to ca. 16.0, while for 2,2,2-trifluoroethanol, it drops to 12.4.^54^ In our case, the electron-withdrawing effect of fluorine atoms is less pronounced due to the quite long distance between the perfluorinated segment and the ionizable group in the molecule. Furthermore, the comparison of the determined p*K*_a_^S^ values demonstrates that the ability of the investigated molecules to undergo dissociation at the water/air boundary increases in the order F_10_H_10_ ≪ F_10_H_10_SH < F_10_H_10_OH ≪ F_10_H_10_COOH. Our results show that effective surface ionization constants for the investigated fluorinated thiol and alcohol are comparable, whereas in bulk, thiols have been reported to be stronger electrolytes than alcohols (for example, bulk p*K*_a_ for ethanol and ethanethiol are equal to 16.0^54^ and 10.9,^55^ respectively); however, this relation has been obtained for hydrogenated molecules.

## 4 Conclusions

Electric surface potential measurements are much less frequently applied for the classical characterization of amphiphiles in monolayers at the free water surface, which is mainly due to difficulties in the interpretation of the measured Δ*V* values. In this paper, a general protocol for estimating particular contributions to a three-layer capacitor model^14^ was developed based on a correlation of the experimentally obtained values with the results from DFT molecular dynamics simulations. The presented methodology has some undeniable advantages in comparison to the previous approaches (for a detailed discussion of previous approaches, see Section 1). First, it can be applied to every amphiphile (even to the surface-active compounds that are devoid of a typical polar group(s) in their structure) as radial distribution functions provide information on the hydration shell. For example, for typical amphiphiles, we found that the hydrated polar part can be considered as −CH_2_OH and not −OH, or −CH_2_SH, and not −SH. Simultaneously, the methyl group in perfluorodecyldecane can also be considered to be hydrated. Second, no assumption of the vertical orientation of molecules at the surface is necessary as molecular dynamics simulations give an average angle between the molecule axis and normal to the water surface. As a result, it becomes possible to correlate the experimental results with the organization, inclination, and average conformation of molecules of interest in the monolayer. Moreover, for ionizable compounds, it has been demonstrated that information on surface dissociation equilibrium is also available. The above-mentioned benefits of combining electrical surface potential measurements with molecular dynamics simulations may be of key importance for the design and initial characterization of molecules for sensor and materials science applications.

## Supporting Information Available

Additional figures presenting the surface pressure–area and electric surface potential change–area isotherms (PDF)

## References

[ref1] Oliveira O. N.; Caseli L.; Ariga K. The Past and the Future of Langmuir and Langmuir–Blodgett Films. Chem. Rev. 2022, 122, 6459–6513. 10.1021/acs.chemrev.1c00754. 35113523

[ref2] Daear W.; Mahadeo M.; Prenner E. J. Applications of Brewster Angle Microscopy from Biological Materials to Biological Systems. Biochim. Biophys. Acta, Biomembr. 2017, 1859, 1749–1766. 10.1016/j.bbamem.2017.06.016. 28655618

[ref3] Sofińska K.; Lupa D.; Chachaj-Brekiesz A.; Czaja M.; Kobierski J.; Seweryn S.; Skirlińska-Nosek K.; Szymonski M.; Wilkosz N.; Wnętrzak A.; Lipiec E. Revealing Local Molecular Distribution, Orientation, Phase Separation, and Formation of Domains in Artificial Lipid Layers: Towards Comprehensive Characterization of Biological Membranes. Adv. Colloid Interface Sci. 2022, 301, 102614 10.1016/j.cis.2022.102614. 35190313

[ref4] Bera P. K.; Kandar A. K.; Kandar A. K.; Krishnaswamy R.; Krishnaswamy R.; Fontaine P.; Impéror-Clerc M.; Pansu B.; Constantin D.; Maiti S.; Sanyal M. K.; Sood A. K. Grazing Incidence X-Ray Diffraction Studies of Lipid-Peptide Mixed Monolayers during Shear Flow. ACS Omega 2020, 5, 14555–14563. 10.1021/acsomega.0c01261. 32596593 PMC7315600

[ref5] Nakahara H.; Nakamura S.; Nakamura K.; Inagaki M.; Aso M.; Higuchi R.; Shibata O. Cerebroside Langmuir Monolayers Originated from the Echinoderms: I. Binary Systems of Cerebrosides and Phospholipids. Colloids Surf., B 2005, 42, 157–174. 10.1016/j.colsurfb.2005.01.012. 15833668

[ref6] Nakahara H.; Minamisono M.; Shibata O. Lateral Interaction of Cholesterol with a Semifluorinated Amphiphile at the Air–Water Interface. Colloids Surf., B 2019, 181, 1035–1040. 10.1016/j.colsurfb.2019.06.072. 31382331

[ref7] Oliveira O. N.; Bonardi C. The Surface Potential of Langmuir Monolayers Revisited. Langmuir 1997, 13, 5920–5924. 10.1021/la970272o. 34736300

[ref8] Nakahara H.; Krafft M. P.; Shibata O. How Self-Assembled Nanodomains Can Impact the Organization of a Phospholipid Monolayer-Flower-Like Arrays. ChemPhysChem 2020, 21, 1966–1970. 10.1002/cphc.202000496. 32710449

[ref9] Wnętrzak A.; Chachaj-Brekiesz A.; Kobierski J.; Karwowska K.; Petelska A. D.; Dynarowicz-Latka P. Unusual Behavior of the Bipolar Molecule 25-Hydroxycholesterol at the Air/Water Interface - Langmuir Monolayer Approach Complemented with Theoretical Calculations. J. Phys. Chem. B 2020, 124, 1104–1114. 10.1021/acs.jpcb.9b10938. 31972080 PMC7497659

[ref10] Ariga K. Don’t Forget Langmuir–Blodgett Films 2020: Interfacial Nanoarchitectonics with Molecules, Materials, and Living Objects. Langmuir 2020, 36, 7158–7180. 10.1021/acs.langmuir.0c01044. 32501699

[ref11] Broniatowski M.; Macho I. S.; Miñones J.; Dynarowicz-Latka P. Langmuir Monolayers Characteristic of (Perfluorodecyl)-Alkanes. J. Phys. Chem. B 2004, 108, 13403–13411. 10.1021/jp0402481.

[ref12] Vogel V.; Möbius D. Local Surface Potentials and Electric Dipole Moments of Lipid Monolayers: Contributions of the Water/Lipid and the Lipid/Air Interfaces. J. Colloid Interface Sci. 1988, 126, 408–420. 10.1016/0021-9797(88)90140-3.

[ref13] Davies J. T.; Rideal S. E. Interfacial Potentials. Can. J. Chem. 1955, 33, 947–960. 10.1139/V55-114.

[ref14] Demchak R. J.; Fort T. Surface Dipole Moments of Close-Packed Un-Ionized Monolayers at the Air-Water Interface. J. Colloid Interface Sci. 1974, 46, 191–202. 10.1016/0021-9797(74)90002-2.

[ref15] Dynarowicz P. Recent Developments in the Modeling of the Monolayers Structure at the Water/Air Interface. Adv. Colloid Interface Sci. 1993, 45, 215–241. 10.1016/0001-8686(93)80029-B.

[ref16] Helmholtz H. Abhandlungen Zur Thermodynamik; Verlag von Wilhelm Engelmann: Leipzig, Germany, 1902.

[ref17] Oliveira O. N.; Taylor D. M.; Lewis T. J.; Salvagno S.; Stirling C. J. M. Estimation of Group Dipole Moments from Surface Potential Measurements on Langmuir Monolayers. J. Chem. Soc., Faraday Trans. 1 1989, 85, 1009–1018. 10.1039/F19898501009.

[ref18] Dynarowicz-Latka P.; Cavalli A.; Silva Filho D. A.; Milart P.; Cristina Dos Santos M.; Oliveira O. N. Quantitative Treatment of Surface Potentials in Langmuir Films from Aromatic Amphiphiles. Chem. Phys. Lett. 2001, 337, 11–17. 10.1016/S0009-2614(01)00175-0.

[ref19] Chachaj-Brekiesz A.; Kobierski J.; Wnȩtrzak A.; Dynarowicz-Latka P. Electrical Properties of Membrane Phospholipids in Langmuir Monolayers. Membranes 2021, 11, 53 10.3390/membranes11010053. 33451035 PMC7828571

[ref20] Volpati D.; Chachaj-Brekiesz A.; Souza A. L.; Rimoli C. V.; Miranda P. B.; Oliveira O. N.; Dynarowicz-Latka P. Semifluorinated Thiols in Langmuir Monolayers – A Study by Nonlinear and Linear Vibrational Spectroscopies. J. Colloid Interface Sci. 2015, 460, 290–302. 10.1016/j.jcis.2015.08.069. 26364075

[ref21] Kissa E. Fluorinated Surfactants and Repellents; Marcel Dekker: New York, 2001.

[ref22] Blanco E.; González-Pérez A.; Ruso J. M.; Pedrido R.; Prieto G.; Sarmiento F. A Comparative Study of the Physicochemical Properties of Perfluorinated and Hydrogenated Amphiphiles. J. Colloid Interface Sci. 2005, 288, 247–260. 10.1016/j.jcis.2005.02.085. 15927586

[ref23] Lo Nostro P. Aggregates from Semifluorinated N-Alkanes: How Incompatibility Determines Self-Assembly. Curr. Opin. Colloid Interface Sci. 2003, 8, 223–226. 10.1016/S1359-0294(03)00052-9.

[ref24] Krafft M. P.; Goldmann M. Monolayers Made from Fluorinated Amphiphiles. Curr. Opin. Colloid Interface Sci. 2003, 8, 243–250. 10.1016/S1359-0294(03)00046-3.

[ref25] Krafft M. P.; Riess J. G. Chemistry, Physical Chemistry, and Uses of Molecular Fluorocarbon-Hydrocarbon Diblocks, Triblocks, and Related Compounds-Unique “Apolar” Components for Self-Assembled Colloid and Interface Engineering. Chem. Rev. 2009, 109, 1714–1792. 10.1021/CR800260K. 19296687

[ref26] Broniatowski M.; Dynarowicz-Latka P. Semifluorinated Alkanes - Primitive Surfactants of Fascinating Properties. Adv. Colloid Interface Sci. 2008, 138, 63–83. 10.1016/j.cis.2007.11.002. 18082155

[ref27] Gaines G. L. Jr. Surface Activity of Semifluorinated Alkanes: F(CF_2_)_m_(CH_2_)_n_H. Langmuir 1991, 7, 3054–3056. 10.1021/la00060A025.

[ref28] Tschierske C. Fluorinated Liquid Crystals: Design of Soft Nanostructures and Increased Complexity of Self-Assembly by Perfluorinated Segments. Top. Curr. Chem. 2012, 318, 1–108. 10.1007/128_2011_267. 22089090

[ref29] Liu X.; Riess J. G.; Krafft M. P. Self-Organization of Semifluorinated Alkanes and Related Compounds at Interfaces: Thin Films, Surface Domains and Two-Dimensional Spherulites. Bull. Chem. Soc. Jpn. 2018, 91, 846–857. 10.1246/bcsj.20170431.

[ref30] Broniatowski M.; Dynarowicz-Latka P.; Witko W. Critical Influence of the Alkane Length in Surface and Liquid–Crystalline Properties of Perfluorodecyl-n-Alkanes. J. Fluorine Chem. 2005, 126, 79–86. 10.1016/j.jfluchem.2004.10.045.

[ref31] Frisch M. J.; Trucks G. W.; Schlegel H. B.; Scuseria G. E.; Robb M. A.; Cheeseman J. R.; Scalmani G.; Barone V.; Petersson G. A.; Nakatsuji H.; Li X.; Caricato M.; Marenich A. V.; Bloino J.; Janesko B. G.; Gomperts R.; Mennucci B.; Hratch D. J. Gaussian 16, Revision B.01; Gaussian, Inc.: Wallingford, CT, 2016.

[ref32] Becke A. D. Density-functional Thermochemistry. III. The Role of Exact Exchange. J. Chem. Phys. 1993, 98, 5648–5652. 10.1063/1.464913.

[ref33] Stephens P. J.; Devlin F. J.; Chabalowski C. F.; Frisch M. J. Ab Initio Calculation of Vibrational Absorption and Circular Dichroism Spectra Using Density Functional Force Fields. J. Phys. Chem. A 1994, 98, 11623–11627. 10.1021/j100096a001.

[ref34] McLean A. D.; Chandler G. S. Contracted Gaussian Basis Sets for Molecular Calculations. I. Second Row Atoms, Z = 11–18. J. Chem. Phys. 1980, 72, 5639–5648. 10.1063/1.438980.

[ref35] Krishnan R.; Binkley J. S.; Seeger R.; Pople J. A. Self-consistent Molecular Orbital Methods. XX. A Basis Set for Correlated Wave Functions. J. Chem. Phys. 1980, 72, 650–654. 10.1063/1.438955.

[ref36] Keith T. A. AIMAll; TK Gristmill Software: Overland Park, KS, 2019.

[ref37] Case D. A.; Cerutti D. S.; Cheatham T. E. I.; Darden T. A.; Duke R. E.; Giese T. J.; Gohlke H.; Goetz A. W.; Greene D.; Homeyer N.; Izadi S.; Kovalenko A.; Lee T. S.; LeGrand S.; Li P.; Lin C.; Liu J.; Luchko T.; Luo R.; Mermelstein D.; Merz K. M.; Monard G.; Nguyen H.; Omelyan I.; Onufriev A.; Pan F.; Qi R.; Roe D. R.; Roitberg A.; Sagui C.; Simmerling C. L.; Botello-Smith W. M.; Swails J.; Walker R. C.; Wang J.; Wolf R. M.; Wu X.; Xiao L.; York D. M.; Kollman P. A. Amber20; University of California: San Francisco, 2021.

[ref38] Martínez L.; Andrade R.; Birgin E. G.; Martínez J. M. PACKMOL: A Package for Building Initial Configurations for Molecular Dynamics Simulations. J. Comput. Chem. 2009, 30, 2157–2164. 10.1002/jcc.21224. 19229944

[ref39] Jorgensen W. L.; Chandrasekhar J.; Madura J. D.; Impey R. W.; Klein M. L. Comparison of Simple Potential Functions for Simulating Liquid Water. J. Chem. Phys. 1983, 79, 926–935. 10.1063/1.445869.

[ref40] Roe D. R.; Cheatham T. E. PTRAJ and CPPTRAJ: Software for Processing and Analysis of Molecular Dynamics Trajectory Data. J. Chem. Theory Comput. 2013, 9, 3084–3095. 10.1021/ct400341p. 26583988

[ref41] Davies J. T.; Rideal E. K. Interfacial Phenomena; Academic Press: New York, 1963.

[ref42] Taylor D. M.; Oliveira O. N.; Morgan H. The Surface Potential of Monolayers Formed on Weak Acidic Electrolytes: Implications for Lateral Conduction. Chem. Phys. Lett. 1989, 161, 147–150. 10.1016/0009-2614(89)85047-X.

[ref43] Morgan H.; Taylor M.; Oliveira O. N. Proton Transport at the Monolayer-Water Interface. Biochim. Biophys. Acta, Biomembr. 1991, 1062, 149–156. 10.1016/0005-2736(91)90386-M. 1848448

[ref44] Dynarowicz-Lątka P.; Cavalli A.; Silva Filho D. A.; dos Santos M. C.; Oliveira O. N. Dipole Moments in Langmuir Monolayers from Aromatic Carboxylic Acids. Chem. Phys. Lett. 2000, 326, 39–44. 10.1016/S0009-2614(00)00726-0.

[ref45] Oliveira O. N. Jr.; Riul A.; Leite V. B. P. Water at Interfaces and Its Influence on the Electrical Properties of Adsorbed Films. Braz. J. Phys. 2004, 34, 73–83. 10.1590/s0103-97332004000100011.

[ref46] Dynarowicz-Latka P.; Cavalli A.; Oliveira O. N. Dissociation Constants of Aromatic Carboxylic Acids Spread at the Air/Water Interface. Thin Solid Films 2000, 360, 261–267. 10.1016/S0040-6090(99)00889-5.

[ref47] Adrien A.; Serjeant E. P. Ionization Constants of Acids and Bases: A Laboratory Manual, 1st ed.; John Wiley & Sons, Inc.: New York, 1962.

[ref48] Kundu S.; Langevin D. Fatty Acid Monolayer Dissociation and Collapse: Effect of PH and Cations. Colloids Surf., A 2008, 325, 81–85. 10.1016/j.colsurfa.2008.04.037.

[ref49] Luo M.; Wauer N. A.; Angle K. J.; Dommer A. C.; Song M.; Nowak C. M.; Amaro R. E.; Grassian V. H. Insights into the Behavior of Nonanoic Acid and Its Conjugate Base at the Air/Water Interface through a Combined Experimental and Theoretical Approach. Chem. Sci. 2020, 11, 10647–10656. 10.1039/d0sc02354j. 33144932 PMC7583472

[ref50] Badban S.; Hyde A. E.; Phan C. M. Hydrophilicity of Nonanoic Acid and Its Conjugate Base at the Air/Water Interface. ACS Omega 2017, 2, 5565–5573. 10.1021/acsomega.7b00960. 31457822 PMC6644816

[ref51] Betts J. J.; Pethica B. A. The Ionization Characteristics of Monolayers of Weak Acids and Bases. Trans. Faraday Soc. 1956, 52, 1581–1589. 10.1039/TF9565201581.

[ref52] Zhao X.; Ong S.; Wang H.; Eisenthal K. B. New Method for Determination of Surface PKa Using Second Harmonic Generation. Chem. Phys. Lett. 1993, 214, 203–207. 10.1016/0009-2614(93)90082-C.

[ref53] Zhao X.; Subrahmanyan S.; Eisenthal K. B. Determination of PKa at the Air/Water Interface by Second Harmonic Generation. Chem. Phys. Lett. 1990, 171, 558–562. 10.1016/0009-2614(90)85263-C.

[ref54] Ballinger P.; Long F. A. Acid Ionization Constants of Alcohols. II. Acidities of Some Substituted Methanols and Related Compounds. J. Am. Chem. Soc. 1960, 82, 795–798. 10.1021/ja01489a008.

[ref55] Kreevoy M. M.; Harper E. T.; Duvall R. E.; Wilgus H. S.; Ditsch L. T. Inductive Effects on the Acid Dissociation Constants of Mercaptans. J. Am. Chem. Soc. 1960, 82, 4899–4902. 10.1021/ja01503a037.

