# Supplementary material for: Comprehensive Approach to the Interpretation of the Electrical Properties of Film-Forming Molecules
Source: J Phys Chem B. 2022 Sep 2;126(36):7037–46. doi: 10.1021/acs.jpcb.2c04526 (PMC9483982; doi:10.1021/acs.jpcb.2c04526)
Supplement: Supplementary file 1 — jp2c04526_si_001.pdf [file jp2c04526_si_001.pdf]

Supporting Information

for

Comprehensive Approach to the Interpretation of the Electrical Properties of Film–Forming Molecules

Anna Chachaj–Brekiesz[*,†], Jan Kobierski[‡], Rosa Griñón Echaniz[†,§], Anita Wnętrzak[†] and Patrycja Dynarowicz–Latka[†]

[†] Department of General Chemistry, Faculty of Chemistry, Jagiellonian University, Gronostajowa 2, 30–387 Kraków, Poland

[‡] Department of Pharmaceutical Biophysics, Faculty of Pharmacy, Jagiellonian University Medical College, Medyczna 9, 30–688 Kraków, Poland

* Email: anna.chachaj@uj.edu.pl

[§] On leave from the Department of Physical Chemistry, University of Zaragoza, C. de Pedro Cerbuna, 12, 50009 Zaragoza, under the Erasmus program

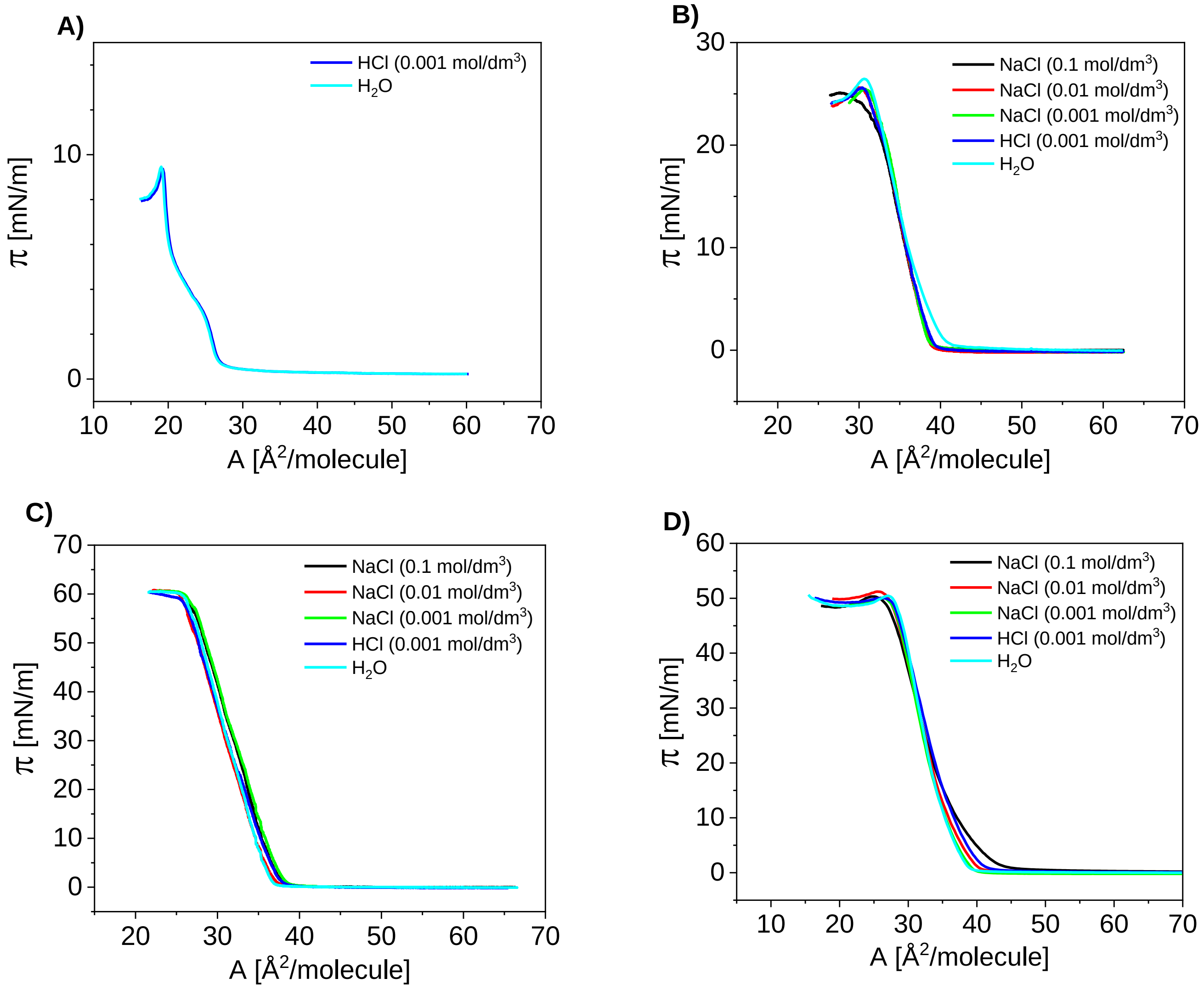

Figure S1. Surface pressure-area isotherms measured for (A) $F_{10}H_{10}$, (B) $F_{10}H_{10}SH$, (C) $F_{10}H_{10}OH$ and (D) $F_{10}H_{10}COOH$ on various subphases at 20°C.

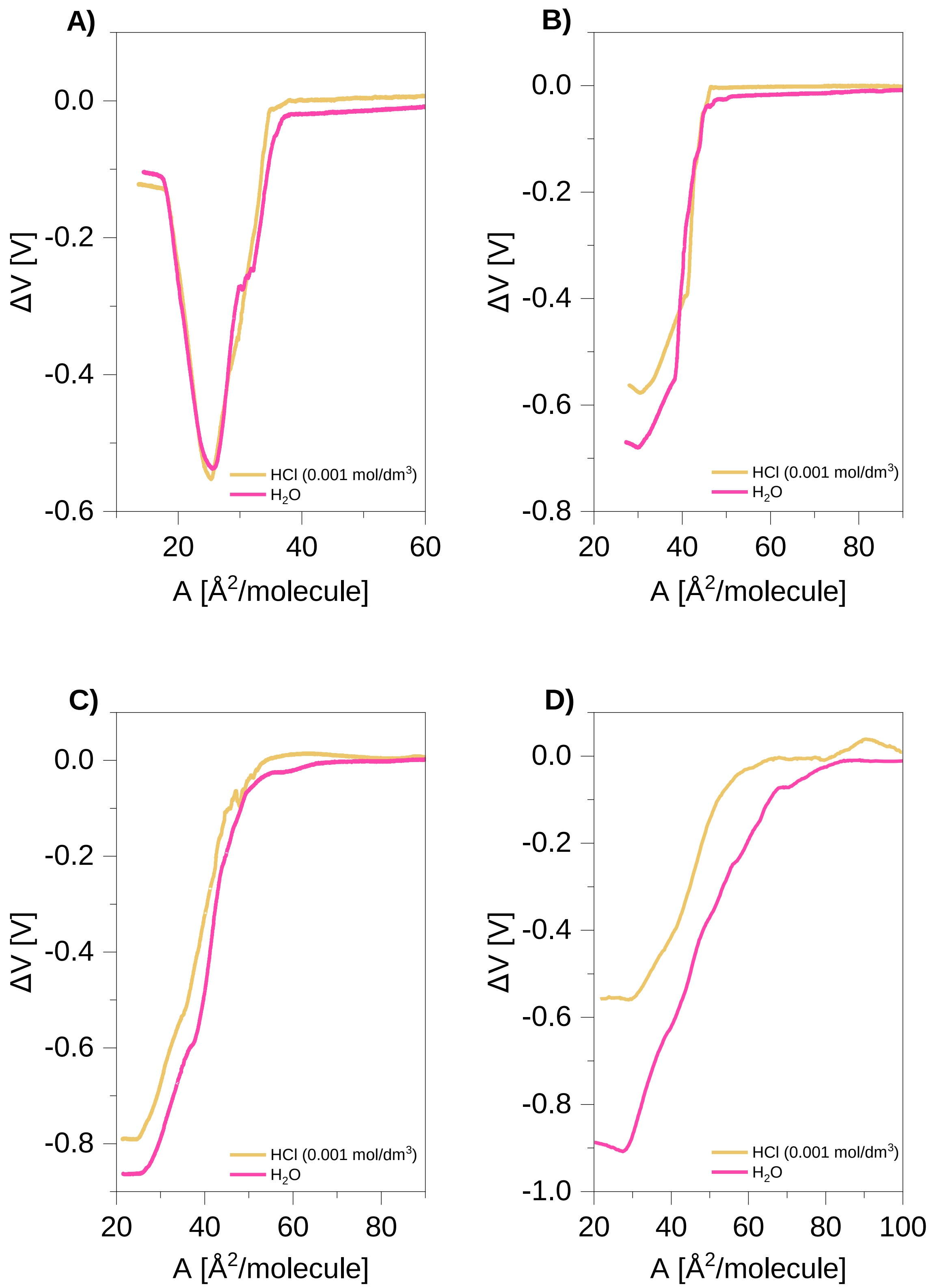

Figure S2. Electric surface potential change-area isotherms measured for (A) $F_{10}H_{10}$, (B) $F_{10}H_{10}SH$, (C) $F_{10}H_{10}OH$ and (D) $F_{10}H_{10}COOH$ on various subphases at 20°C.
